# 6-Methoxyflavanones as Bitter Taste Receptor Blockers for hTAS2R39

**DOI:** 10.1371/journal.pone.0094451

**Published:** 2014-04-10

**Authors:** Wibke S. U. Roland, Robin J. Gouka, Harry Gruppen, Marianne Driesse, Leo van Buren, Gerrit Smit, Jean-Paul Vincken

**Affiliations:** 1 Laboratory of Food Chemistry, Wageningen University, Wageningen, The Netherlands; 2 Unilever R&D, Vlaardingen, The Netherlands; Monell Chemical Senses Center, United States of America

## Abstract

Many (dietary) bitter compounds, *e.g*. flavonoids, activate bitter receptor hTAS2R39 in cell-based assays. Several flavonoids, amongst which some flavanones, are known not to activate this receptor. As certain flavanones are known to mask bitter taste sensorially, flavanones might act as bitter receptor antagonists. Fourteen flavanones were investigated for their potential to reduce activation of hTAS2R39 by epicatechin gallate (ECG), one of the main bitter compounds occurring in green tea. Three flavanones showed inhibitory behavior towards the activation of hTAS2R39 by ECG: 4′-fluoro-6-methoxyflavanone, 6,3′-dimethoxyflavanone, and 6-methoxyflavanone (in order of decreasing potency). The 6-methoxyflavanones also inhibited activation of hTAS2R14 (another bitter receptor activated by ECG), though to a lesser extent. Dose-response curves of ECG at various concentrations of the full antagonist 4′-fluoro-6-methoxyflavanone and wash-out experiments indicated reversible insurmountable antagonism. The same effect was observed for the structurally different agonist denatonium benzoate.

## Introduction

Even though bitter taste can be appreciated in some food products, such as beer, coffee, dark chocolate and red wine [1], in most cases bitterness in food is unwanted and efforts are taken to reduce bitter taste [2]. One approach for masking bitter taste is the use of so-called bitter receptor blockers, which inhibit the taste receptor activation caused by the bitter compound. On the human tongue, bitterness is perceived by human bitter taste receptors (hTAS2Rs, TAS2Rs or T2Rs). The *in-vitro* activation of these hTAS2Rs by bitter compounds has been studied intensively during the last decade. For 21 of the 25 the bitter receptors, an agonist, or in some cases dozens of agonists, have been identified [3], [4]. On the contrary, bitter receptor antagonists are still quite rare.

The small molecule (4-(2,2,3-trimethylcyclopentyl)butanoic acid (or GIV 3727) has been reported as inhibitor of six bitter taste receptors [5]. It was able to decrease the sensory perception of bitter aftertaste of the sweeteners acesulfame K and saccharin, as well as the activation of hTAS2R31 and hTAS2R43, the bitter receptors activated by these two compounds. Another compound, the decreased bitter receptor activation of which could be linked to sensory perception, was *p*-(dipropylsulfamyl)benzoic acid (better known as probenecid). It has been reported to inhibit activation of hTAS2R16, hTAS2R38, and hTAS2R43, and to suppress the bitter taste perception of salicin in sensory tests [6]. It has been reported that a compound can act as an agonist towards one subset of bitter receptors, whereas it can act as an antagonist towards another subset of bitter receptors. This has been described for the two sesquiterpene lactones 3β-hydroxydihydrocostunolide (3HDC) and 3β-hydroxypelenolide (3HP) [7].

Recently, a pharmacophore model for maskers of the bitter taste of caffeine has been developed [8]. This pharmacophore was docked into a homology model of hTAS2R10 (one of the bitter receptors activated by caffeine). Docking of the two substances enterolactone and enterodiol predicted their bitterness modulating activities, which could be confirmed by sensory tests. Docking was also applied for the compound GIV 3727 in a model of hTAS2R31, and the presence of a single binding pocket was reasoned [5], in which both agonist and antagonist can bind.

Apart from *in-vitro* studies on bitter receptor blocking, several molecules are reported to mask bitter taste *in-vivo* [2], amongst which the flavanones homoeriodictyol, its Na-salt, and eriodictyol. They reduced the bitter taste of different chemical classes of bitter molecules up to 40% with unknown mechanism [9]. Their sensorial bitter masking effect has not been proven to be caused by inhibition of bitter taste receptor activation. Two other flavanones (sakuranetin and 6-methoxysakuranetin) have been described as antagonists for hTAS2R31 [10]. Hence, flavanones seem to be of importance in reduction of bitter taste and bitter taste receptor activation.

The human bitter taste receptor hTAS2R39 seems to be a bitter receptor for dietary compounds, as many agonists are dietary compounds, such as thiamine (vitamin B1), quinine [3] used in tonic water, catechins from green tea [11], wine tannin precursors [12], small peptides from casein hydrolysates [13] and cheese [14], isoflavones from soy bean [15], and many other flavonoids from several plant sources [16]. Hence, it is of interest to identify a bitter blocker for this receptor. It is likely that an antagonist might have similar structural elements to an agonist in order to fit into the same binding pocket. In our previous study on (iso)flavonoid agonists of hTAS2R39, several of the compounds tested, amongst which flavanones, did not activate the bitter receptor despite structural similarity to active compounds [16]. The aim of the present study was to investigate whether these and other flavanones could act as antagonists towards hTAS2R39. It was demonstrated that some flavanones showed antagonistic behavior, while others did not.

## Materials and Methods

### Materials

Compounds tested were obtained from Extrasynthese (Genay, France), Indofine Chemical Company (Hillsborough, NJ, USA), Interbioscreen (Moscow, Russia), and Sigma-Aldrich (Steinheim, Germany). The majority of compounds were ≥99% or ≥98% pure; compound (**4**) was 95% pure and compound (**6**) was 92–95% pure. Each compound was dissolved in DMSO (Sigma-Aldrich) to a 100 mM stock concentration. Trypan blue solution (0.4% w/v) and isoproterenol were purchased from Sigma-Aldrich.

Tyrode's buffer (140 mM NaCl, 5 mM KCl, 10 mM glucose, 1 mM MgCl_2_, 1 mM CaCl_2_, and 20 mM Hepes, pH 7.4) with 0.5 mM probenecid (Sigma-Aldrich) was used for dilution of compound-DMSO stock solutions and for calcium imaging assays. The presence of probenecid in the buffer did not lead to inhibition of hTAS2R14 or hTAS2R39. Comparisons of assays with and without the use of probenecid are shown in **File S1**. All compounds were tested for autofluorescence and toxic effects on the cells (**File S2**) used at a concentration of 1 mM as described before [15].

### Expression of hTAS2R39 and hTAS2R14 in HEK293 cells

For functional expression of the human bitter taste receptor hTAS2R39, HEK293 T-Rex Flp-In cells (Invitrogen, San Diego, CA, USA) were used, stably expressing the chimeric G-protein α-subunit Gα16-gust44 (cloned into pcDNA4 (Invitrogen)) [17] and the human bitter receptor genes for hTAS2R39 (cloned into pcDNA5/FRT (Invitrogen)). The bitter receptor gene contained a DNA sequence encoding the first 45 amino acids of rat somatostatin receptor type 3 at its 5′ end (the receptor expression was achieved according to [18] with exception of the HSV-tag), in order to improve membrane targeting of the receptor protein. The same procedure was applied for stable expression of hTAS2R14. Cells were maintained in Dulbecco's Modified Eagle's Medium (DMEM) and 10% (v/v) tetracycline-free FBS (both Lonza, Verviers, Belgium) supplemented with blasticidin (5 *μ*g/mL), geneticin (400 *μ*g/mL) and hygromycin (100 *μ*g/mL) (all from Invitrogen). Cells were grown and maintained at 37 °C and 5% (v/v) CO_2_.

### *Monitoring bitter receptor activation by intracellular calcium release*

Cells were seeded into poly-L-lysine-coated (Sigma-Aldrich) 96-well plates (black wall, clear bottom, Greiner bio-one, Frickenhausen, Germany) at a density of 7*10^3^ cells in 100 *μ*L/well and cultured for 24 h. Transcription of the receptors was induced by adding 0.25 *μ*g/mL doxycycline (Sigma-Aldrich). Cells were induced for 24 h and then loaded with the calcium-sensitive fluorescent dye Fluo-4-AM (2.5 *μ*M, Invitrogen), which was dissolved in Tyrode's buffer containing 5% (v/v) tetracycline-free FBS (Lonza). One hour after loading, cells were washed with Tyrode's buffer and taken up in Tyrode's buffer. Stock solutions of test compounds were prepared in DMSO and diluted to the appropriate concentration in Tyrode's buffer, not exceeding a DMSO concentration of 1% (v/v).

Receptor activation or inhibition was measured via intracellular Ca^2+^ release [19] in a FlexStation II 384 or FlexStation III (Molecular Devices Corporation, Sunnyvale, CA, USA) by measuring fluorescence (excitation 485 nm/emission 520 nm) for either 90 s or 240 s at 37°C. Two methods of compound administration were applied: simultaneous and stepwise addition of potential antagonist and agonist. The first 17 s before compound addition were used for baseline determination. For the simultaneous method, agonist and potential antagonist were pre-mixed, administered after 17 s, and fluorescence was measured for in total 90 s. For the stepwise method, after 17 s, the potential antagonist was added, fluorescence was measured until 120 s, and after 120 s the agonist was added and measured for another 120 s, in total 240 s. Non-induced cells, which did not express the taste receptor, were measured in parallel to verify specificity of receptor activation. Each experiment was performed at least in duplicate in separate experiments (as indicated for each figure).

### Calcium assay data processing

Data processing was done as reported previously [15]. In brief, SoftMax Pro 5.4 software (Molecular Devices Corporation) was used to plot the fluorescence signals. The fluorescence value (ΔF/F_0_), representing receptor activity, was calculated by subtracting the baseline fluorescence (F_0_) prior to loading from the maximum fluorescence (F) after compound addition, divided by the signal of the baseline in order to normalize background fluorescence [20]. Dose-response curves were established as non-linear regression curves using Graph Pad Prism (version 4 for Windows, Graph Pad Software, San Diego, CA, USA). Half-maximal effective concentrations (EC_50_) and half-maximal inhibitory concentrations (IC_50_) were calculated. Error bars reflect the standard error of the mean (SEM). Statistical analysis was performed in Graph Pad Prism (one-way ANOVA at 5% risk level, followed by Bonferroni's post hoc test).

### Investigation of inhibitory behavior of flavanones

Measuring dose-response curves of ECG on hTAS2R39 under the conditions used in this study revealed an EC_80_ concentration of 200 μM. Screening for hTAS2R39 inhibition by flavanones was performed with simultaneous application of agonist (200 μM ECG) and putative antagonist (250 μM flavanone). Inhibition was indicated when 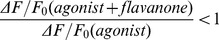. In case of indicated inhibition, flavanones were applied at different concentrations, in order to test for dose-dependent inhibition, at the EC_80_ concentration of the agonist. Another agonist of hTAS2R39, denatonium benzoate, was used in inhibition experiments at 1.7 mM (EC_80_). hTAS2R14 inhibition was tested with 640 μM ECG or 70 μM genistein as agonists, representing their respective EC_80_ concentrations. To investigate the specificity of flavanones for inhibition of hTAS2R39 and hTAS2R14, another bitter taste receptor, hTAS2R16 (not known to be activated by flavonoids), was used in inhibition experiments (**File S3**) towards its agonist salicin.

To determine whether inhibition was specific for the bitter taste receptor, the effect of the antagonists was tested on the β2-adrenergic receptor agonist isoproterenol (50 μM). To this end, the antagonists were applied at ∼IC_50_ concentrations (100 μM 4′-fluoro-6-methoxyflavanone, 500 μM 6,3′-dimethoxyflavanone, and 500 μM 6-methoxyflavanone). To distinguish between reversible and irreversible inhibition, washout experiments were performed. Cells were stimulated with 200 μM ECG in the absence and in the presence of each antagonist (∼IC_50_ concentrations), washed with Tyrode's buffer (80 *μ*L/well), and again stimulated with 200 μM ECG.

## Results

### Identification and characterization of hTAS2R39 inhibitors

**Figure 1:**
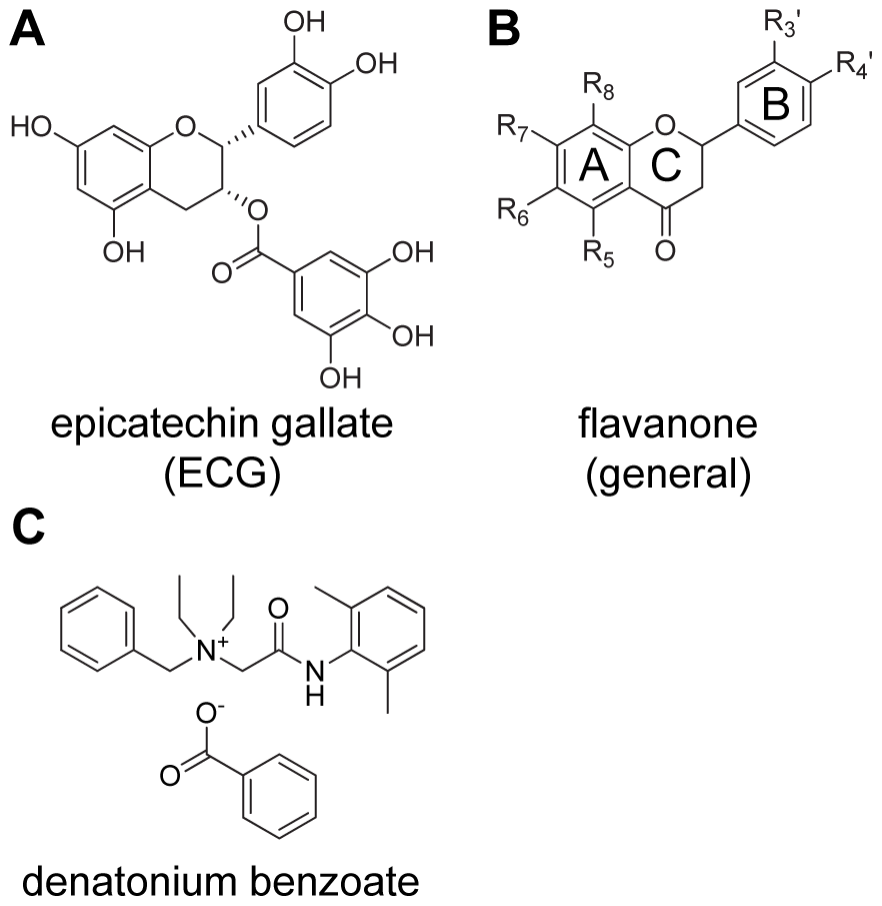
Chemical structures of hTAS2R39 agonist epicatechin gallate (ECG) (A), investigated flavanones (residues specified in Table 1) (B) and hTAS2R39 agonist denatonium benzoate (C). The commonly applied ring-nomenclature for flavonoids (A-, B-, and C-ring) is shown in the general structure formula for flavanones.

**Figure 2:**
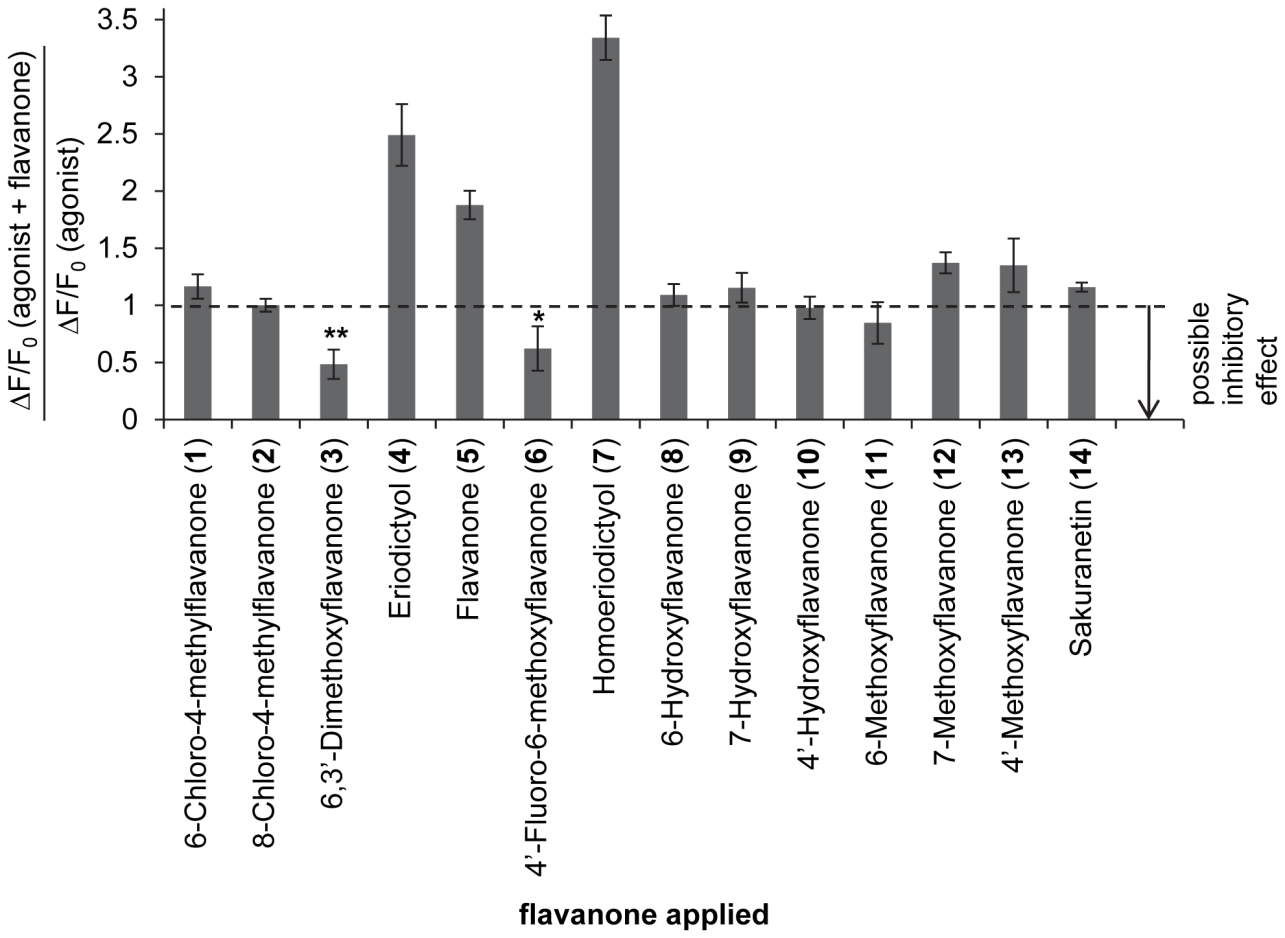
Screening of flavanones for reduction of ECG response on hTAS2R39. Screening was performed with simultaneous application of agonist (200 μM ECG) and putative antagonist (250 μM flavanone). Inhibition was indicated when 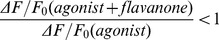. Data are presented as mean ±SD of n separate experiments conducted in duplicate. Compounds **1**, **2**, **4**, **5**, **7**, **10**: n = 2, compounds **8**, **9**, **12**, **13**, **14**: n = 3, compound **3**: n = 4, compound **6**, **11**: n = 5. Significance of signal reduction is indicated by ** (p≤0.01) and * (p≤0.05).

Epicatechin gallate (ECG) (**Figure 1A**), one of the main bitter compounds in green tea [11], was chosen as agonist of hTAS2R39. In a previous study we identified nine flavanones as agonists of hTAS2R39, whereas three other flavanones did not activate this receptor [16]. As some flavanones have been reported as bitter blockers [9], [10], it was investigated whether the three inactive flavanones, as well as other flavanones, might have antagonistic properties towards hTAS2R39. Fourteen flavanones (**Table 1** **and** **Figure 1B**) were screened for their ability to reduce the activation of hTAS2R39 by ECG. Inhibition was indicated when the ratio between ECG response in the presence of a flavanone and the ECG response in the absence of a flavanone was <1 (**Figure 2**). In this figure, it can be seen that three compounds showed reduction of ECG responses on hTAS2R39: 6,3′-dimethoxyflavanone (**3**), 4′-fluoro-6-methoxyflavanone (**6**), and 6-methoxyflavanone (**11**). The inhibitory effects of (**3**) and (**6**) at screening concentrations were significant. The effect of (**11**) was not significant, but as there was a trend of reduced ECG responses visible, also (**11**) was selected for investigation of dose-response behavior.

**Table 1:** Flavanones tested for reduction of activation of hTAS2R39 by ECG.

	R_5_	R_6_	R_7_	R_8_	R_3′_	R_4′_
6-Chloro-4-methylflavanone (**1**)	H	Cl	H	H	H	CH_3_
8-Chloro-4-methylflavanone (**2**)	H	H	H	Cl	H	CH_3_
6,3′-Dimethoxyflavanone (**3**)	H	OCH_3_	H	H	OCH_3_	H
Eriodictyol^a^ (**4**)	OH	H	OH	H	OH	OH
Flavanone^b^ (**5**)	H	H	H	H	H	H
4′-Fluoro-6-methoxyflavanone (**6**)	H	OCH_3_	H	H	H	F
Homoeriodictyol^a^ (**7**)	OH	H	OH	H	OCH_3_	OH
6-Hydroxyflavanone (**8**)	H	OH	H	H	H	H
7-Hydroxyflavanone (**9**)	H	H	OH	H	H	H
4′-Hydroxyflavanone^b^ (**10**)	H	H	H	H	H	OH
6-Methoxyflavanone^b^ (**11**)	H	OCH_3_	H	H	H	H
7-Methoxyflavanone (**12**)	H	H	OCH_3_	H	H	H
4′-Methoxyflavanone (**13**)	H	H	H	H	H	OCH_3_
Sakuranetin^c^ (**14**)	OH	H	OCH_3_	H	H	OH

Residues relate to **Figure 1B**.

a activated hTAS2R39 in a previous study [16], but was selected for testing as antagonist due to ability to reduce bitter taste perception in sensory tests [9].

b no activation of hTAS2R39 in a previous study [16].

c reported as antagonist of hTAS2R31 [10].

**Figure 3:**
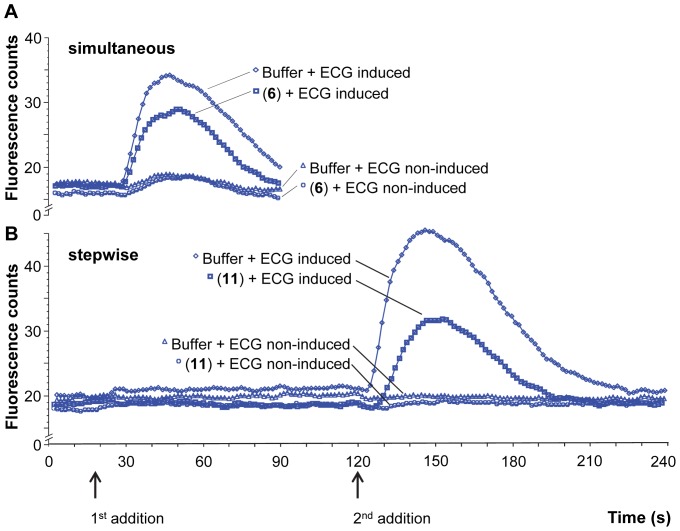
Fluorescent counts of ECG-induced calcium responses in cells expressing hTAS2R39 (induced) and non-expressing hTAS2R39 (non-induced). **A**: Simultaneous addition of agonist (ECG 200 μM) and antagonist (4′-fluoro-6-methoxyflavanone (**6**) 16 μM) (□) versus the signal elicited by ECG (antagonist replaced by buffer, final concentration ECG 200 μM) (◊). Non-induced cells: ECG 200 μM and 4′-fluoro-6-methoxyflavanone (**6**) 16 μM (○) and ECG 200 μM (Δ). **B**: Stepwise addition of first antagonist (arrow “1^st^ addition”), and then agonist (arrow “2^nd^ addition”) (1^st^ 6-methoxyflavanone (**11**) 500 μM, 2^nd^ ECG 200 μM) (□)) versus agonist (1^st^ buffer, 2^nd^ ECG 200 μM) (◊). Non-induced cells: 1^st^ 6-methoxyflavanone (**11**) 500 μM, 2^nd^ ECG 200 μM (○) versus 1^st^ buffer, 2^nd^ ECG 200 μM (Δ).

They were further investigated by two different ways of compound addition: simultaneous and stepwise addition. Simultaneous addition of agonist and antagonist to the receptor reflects the situation of ideal blocker application in food products. Stepwise addition of agonist and antagonist is commonly applied in pharmaceutical research when examining pharmacodynamics of receptor-antagonist interaction [21]. **Figure 3** illustrates the different ways of compound administration for representative examples. **Figure 3A** shows simultaneous addition of agonist and antagonist, which implies that after baseline fluorescence measurement, agonist and antagonist were added simultaneously (indicated with the arrow “1^st^ addition”). **Figure 3B** shows stepwise addition of agonist and antagonist, which implies that after baseline fluorescence measurement, first the antagonist (or buffer) was added (indicated with the arrow “1^st^ addition”), and subsequently the agonist was added (indicated with the arrow “2^nd^ addition”). In both examples, calcium signals elicited by ECG decreased in the presence of the 6-methoxyflavanones.

**Figure 4:**
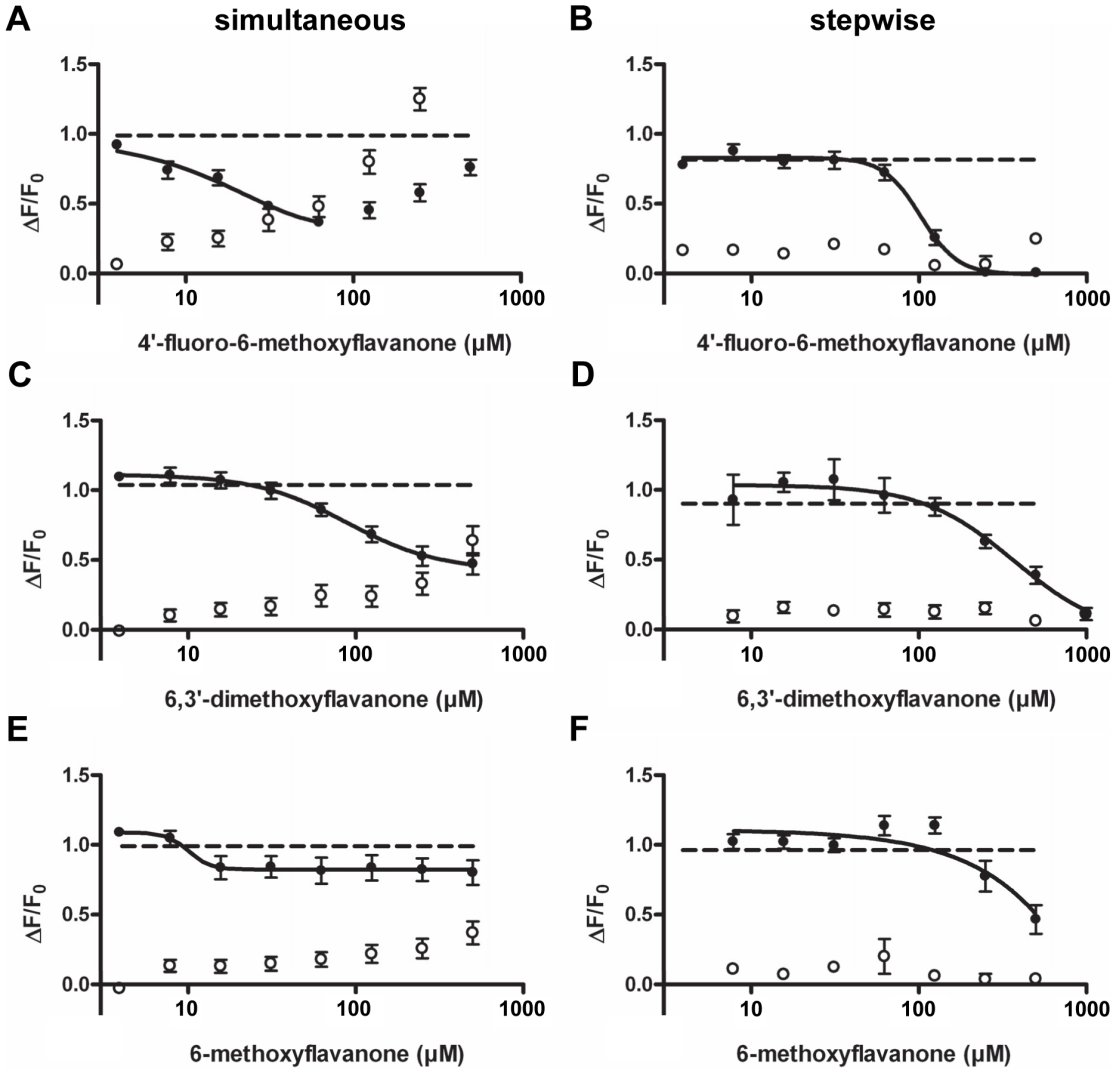
Inhibition of response of 200 μM ECG (---) on hTAS2R39 (induced (•), non-induced (○)) by 4′-fluoro-6-methoxyflavanone (**6**) after simultaneous addition (n = 5) (**A**) and stepwise addition (n = 4) (**B**), by 6,3′-dimethoxyflavanone (**3**) after simultaneous addition (n = 4) (**C**) and stepwise addition (n = 3) (**D**), and by 6-methoxyflavanone (**11**) after simultaneous addition (n = 5) (**E**) and stepwise addition (n = 3) (**F**). Data are presented as mean ±SEM of n separate experiments conducted in duplicate.

The inhibitory properties of the compounds 6,3′-dimethoxyflavanone (**3**), 4′-fluoro-6-methoxyflavanone (**6**), and 6-methoxyflavanone (**11**) towards hTAS2R39 were investigated using both ways of compound addition. The compound 4′-fluoro-6-methoxyflavanone (**6**) showed inhibitory activity towards ECG on hTAS2R39 both after simultaneous (**Figure 4A**) and after stepwise addition (**Figure 4B**). Application of (**6**) prior to addition of ECG led to 100% receptor blocking (**Figure 4B**). For this full receptor blocker, the half-maximal inhibitory concentration (IC_50_) was 102 μM. An overview of inhibition thresholds and IC_50_ values is given in **Table 2**. When (**6**) was added simultaneously with ECG, it had a lower inhibition threshold than when added in the stepwise way. Upon simultaneous addition, a maximal signal reduction of 65% was reached at 63 μM, and further signal reduction could not be observed due to non-specific signals of the compound itself. The same holds for the simultaneous addition of 6,3-dimethoxyflavanone (**3**) (**Figure 4C**), where a maximal signal reduction of 55% was reached at 500 μM. Due to increasing non-specific signals of (**3**), the full efficacy upon simultaneous addition could not be established. As shown in **Figure 4D**, a maximal reduction of ∼85% at 1000 μM was reached by (**3**) after stepwise addition. In contrast to (**6**) and (**3**), the compound 6-methoxyflavanone (**11**) showed negligible inhibitory activity against ECG on hTAS2R39 when applied simultaneously (**Figure 4E**), whereas it showed inhibitory activity when applied stepwise (ca. 50% reduction of activation at 500 μM) (**Figure 4F**). When investigating the inhibitory behavior of the compounds identified, it thus became clear that the way of antagonist addition influenced the efficacy of the antagonist.

**Table 2:** Thresholds and IC_50_ values of 6-methoxyflavanones for inhibition of hTAS2R39 responses towards 200 μM ECG and 1.7 mM denatonium benzoate.

hTAS2R39 agonists		Flavanones	Simultaneous		Stepwise	
ECG	Denatonium		Threshold	IC_50_	Threshold	IC_50_
(μM)	(mM)		(μM)	(μM)	(μM)	(μM)
200	0	6-methoxyflavanone	n.b.	n.b.	250	479±199
200	0	6,3-dimethoxyflavanone	63	282±97	125	407±55
200	0	4′-fluoro-6-methoxyflavanone	8	32±7	63	102±9
0	1.7	6-methoxyflavanone	n.b.	n.b.	500	n.d.
0	1.7	6,3-dimethoxyflavanone	8	89±6	63	240±8
0	1.7	4′-fluoro-6-methoxyflavanone	8	22±12	32	55±3

n.b., no blocking.

n.d., not determined.

**Figure 5:**
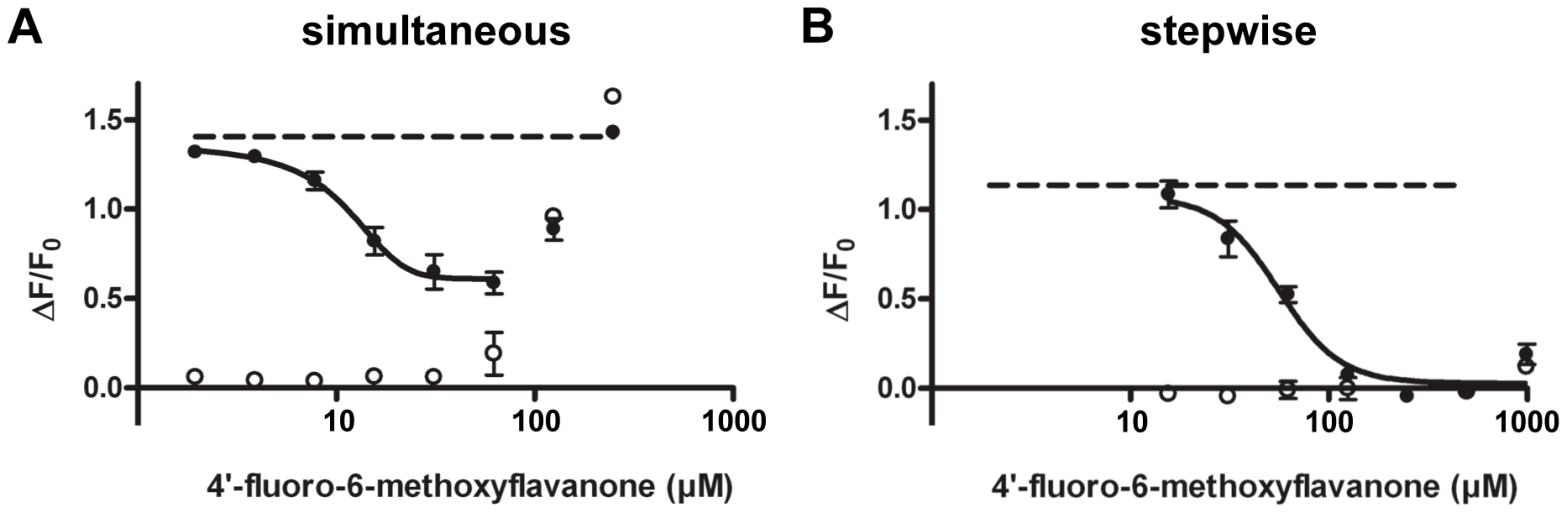
Inhibition of response of 1.7(---) on hTAS2R39 (induced (•), non-induced (○)) by 4′-fluoro-6-methoxyflavanone (6) after simultaneous addition (n = 4) (A) and stepwise addition (n = 4) (B). Data are presented as mean ±SEM of n separate experiments conducted in duplicate.

Next, it was investigated whether the activation of hTAS2R39 by another agonist could also be reduced by the inhibitors identified. Denatonium benzoate (**Figure 1C**) was selected as well known agonist of hTAS2R39, which is different from ECG, in terms of structure and activation concentrations. Dose-response curves of denatonium benzoate were measured, and an EC_50_ of 711 μM, and an EC_80_ of 1.7 mM were established (data not shown). **Figure 5** shows the inhibitory behavior of 4′-fluoro-6-methoxyflavanone (**6**) towards 1.7 mM denatonium benzoate on hTAS2R39. The same trends as for ECG combined with antagonists were observed. The receptor activation of denatonium benzoate was reduced upon simultaneous addition of (**6**) up to a concentration of 63 μM (not further reduced due to increasing non-specific signals), leading to maximal signal reduction of 58%. Stepwise application of (**6**) led to 100% receptor blocking, as already seen with ECG. The IC_50_ was calculated to be 55 μM. The results of all antagonists applied with denatonium benzoate are summarized in **Table 2**. Due to the fact that structurally different agonists were inhibited in a similar manner, we conclude that the reduced receptor response was not achieved by interaction between agonist and inhibitor, but by actual receptor antagonism.

### Specificity of hTAS2R39 inhibitors

**Figure 6:**
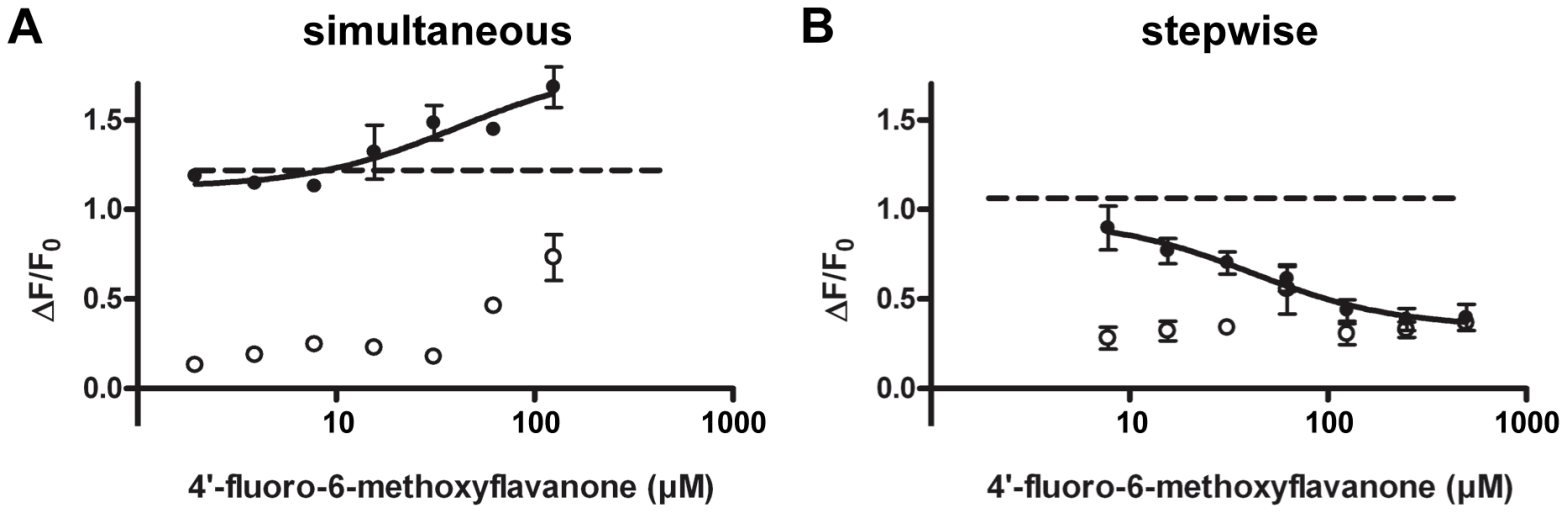
Inhibition of response of 640 μM ECG (---) on hTAS2R14 (induced (•), non-induced (○)) by 4′-fluoro-6-methoxyflavanone (6) after simultaneous addition (n = 2) (A) and stepwise addition (n = 3) (B). Data are presented as mean ±SEM of n separate experiments conducted in duplicate.

It was investigated whether the antagonists identified specifically inhibit hTAS2R39, or also hTAS2R14, as many flavonoids behave similarly towards these two receptors [16]. As agonist for hTAS2R14, ECG was used. **Figure 6** shows that no blocking of hTAS2R14 occurred upon simultaneous application of 4′-fluoro-6-methoxyflavanone (**6**) and ECG (here at 640 μM, EC_80_ on hTAS2R14). The increase of signal at increasing concentrations of (**6**) is of non-specific nature, which can also be seen in the increase of response of non-induced cells, in which the bitter receptor is not expressed. Upon stepwise application, an inhibitory effect was observed. The three methoxyflavanones were also tested with genistein, another agonist of hTAS2R14, at 70 μM (EC_80_). The results were similar to the results obtained with ECG. An overview of inhibition thresholds and IC_50_ values on hTAS2R14 is given in **Table 3**. It is remarkable that none of the three methoxyflavanones blocked hTAS2R14, when applied simultaneously with one of the agonists.

**Table 3:** Thresholds and IC_50_ values of 6-methoxyflavanones for inhibition of hTAS2R14 responses towards 640 μM ECG and 70 μM genistein.

hTAS2R14 agonists		Flavanones	Simultaneous		Stepwise	
ECG	Genistein		Threshold	IC_50_	Threshold	IC_50_
(μM)	(μM)		(μM)	(μM)	(μM)	(μM)
640	0	6-methoxyflavanone	n.b.	n.b.	250	447±123
640	0	6,3-dimethoxyflavanone	n.b.	n.b.	125	∼250
640	0	4′-fluoro-6-methoxyflavanone	n.b.	n.b.	<8	79±25
0	70	6-methoxyflavanone	n.b.	n.b.	500	741±143
0	70	6,3-dimethoxyflavanone	n.b.	n.b.	n.d.	n.d.
0	70	4′-fluoro-6-methoxyflavanone	n.b.	n.b.	32	∼500

n.b., no blocking.

n.d., not determined.

**Figure 7:**
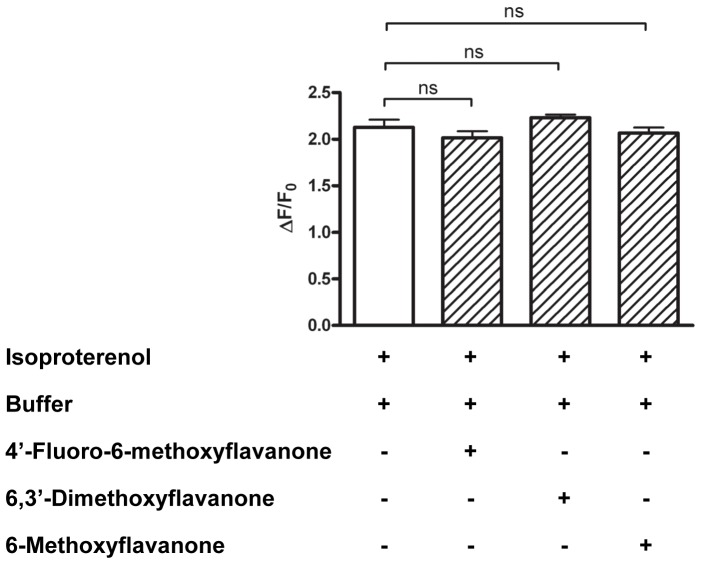
Isoproterenol responses upon application with buffer, 4′-fluoro-6-methoxyflavanone (6), 6,3′-dimethoxyflavanone (3), and 6-methoxyflavanone (11). Data are presented as mean ±SEM of a representative experiment conducted in quadruplicate. n.s., not significant.

In order to further investigate the specificity of the antagonists identified towards taste receptors, isoproterenol responses were measured. Inhibition of isoproterenol response would indicate non-specific inhibition of β2-adrenergic receptors, endogenous to HEK293 cells. The results (**Figure 7**) show that the isoproterenol responses were not reduced and therewith the inhibition is concluded to be specific for taste receptors. Furthermore, the specificity of the antagonists was further investigated by measuring their activity on another bitter taste receptor, hTAS2R16. No blocking of salicin responses on hTAS2R16 was observed, neither during simultaneous, nor stepwise addition (**File S3**).

### Pharmacological characterization of 4′-fluoro-6-methoxyflavanone

**Figure 8:**
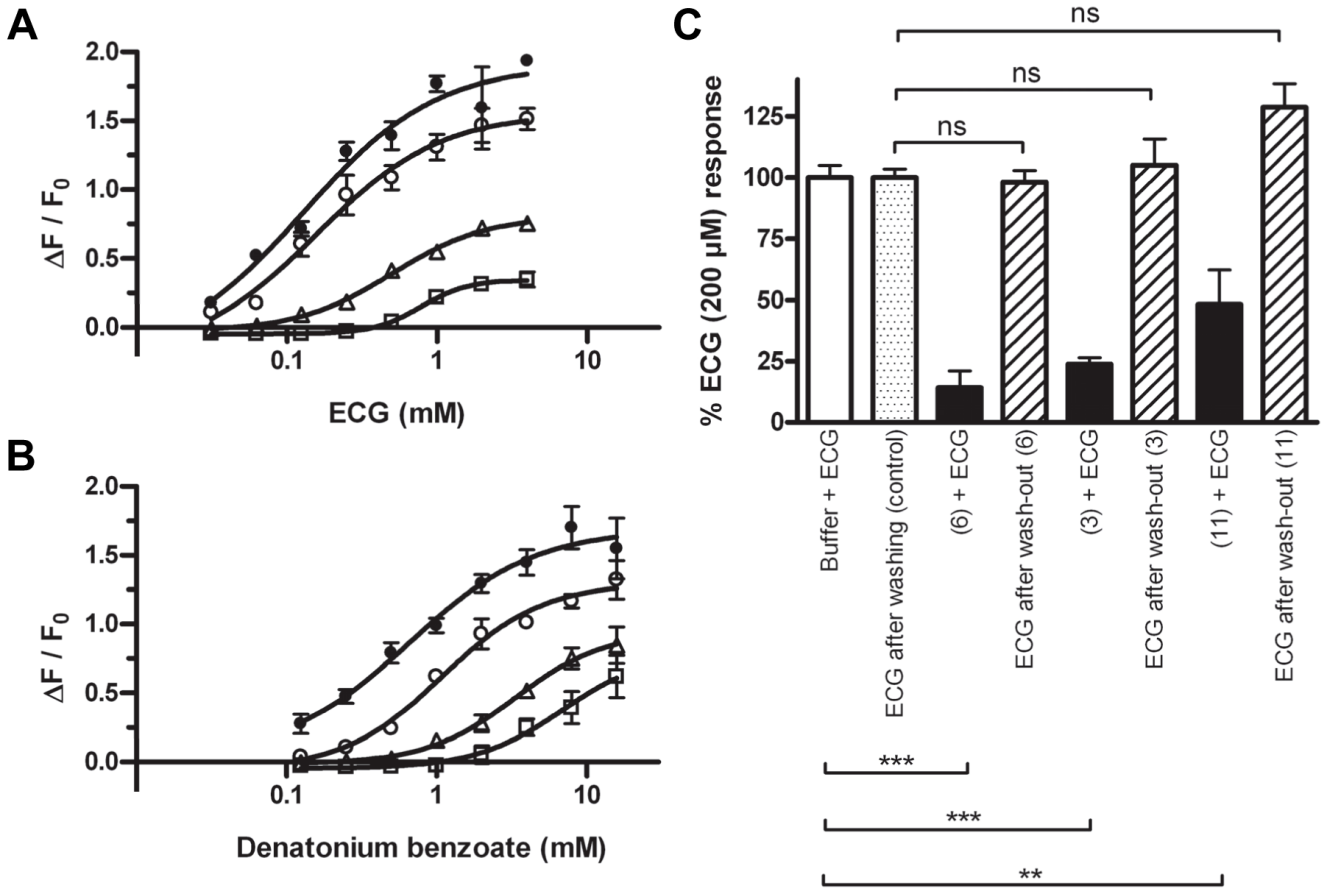
Dose-response curves for epicatechin gallate (ECG) (•) (n = 2) (A) and denatonium benzoate (•) (n = 2) (B) on hTAS2R39, and their modification by increasing 4′-fluoro-6-methoxyflavanone (6) concentrations (○ 50 μM, Δ 100 μM, □ 200 μM). Antagonist and agonist were added in the stepwise way. Data are presented as mean ±SEM of n separate experiments conducted in duplicate. Wash-out experiments (n = 2) (C). Cells were stimulated with 200 μM ECG in the absence (open bars) and in the presence (filled bars) of 100 μM 4′-fluoro-6-methoxyflavanone (6), or 500 μM 6,3′-dimethoxyflavanone (3), or 500 μM 6-methoxyflavanone (11), washed with Tyrode's buffer, and again stimulated with 200 μM ECG (hatched bars; control: grey bar). Antagonist and agonist were added in the stepwise way. Data are presented as mean ±SEM of n separate experiments conducted in quadruplicate. Significance of signal reduction is indicated by *** (p≤0.001), ** (p≤0.01), and n.s. (not significant, p>0.05).

Due to full elimination of agonistic responses by 4′-fluoro-6-methoxyflavanone (**6**) on hTAS2R39, this molecule seemed to be the most effective antagonist identified in this study. The mechanism of antagonism was further clarified by measuring dose-response curves of ECG (**Figure 8A**) and denatonium benzoate (**Figure 8B**) in the presence of various concentrations of (**6**). Two effects were observed upon increasing antagonist concentrations: the dose-response curves shifted to the right, and the signal amplitudes decreased. The dose-response curves for the inhibition of ECG and denatonium benzoate showed the same pattern. EC_50_ values at all antagonist concentrations were calculated and are given in **Table 4**.

**Table 4:** EC_50_ values of ECG and denatonium benzoate in the presence of various concentrations of 4′-fluoro-6-methoxyflavanone (**6**) on hTAS2R39.

	Concentration of 4′-fluoro-6-methoxyflavanone			
	0 μM	50 μM	100 μM	200 μM
ECG	128±82 μM	156±63 μM	501±48 μM	781±93 μM
Denatonium benzoate	659±334 μM	1.09±0.21 mM	3.36±0.84 mM	6.94±4.98 mM

To distinguish between reversible and irreversible inhibition, washout experiments were performed. In **Figure 8C** it can clearly be seen that the inhibition was reversible.

## Discussion

In this paper we describe, to our knowledge for the first time, the identification of antagonists for hTAS2R39. For hTAS2R39, 4′-fluoro-6-methoxyflavanone (**6**), 6,3′-dimethoxyflavanone (**3**), and 6-methoxyflavanone (**11**) were identified as antagonists (in decreasing order of potency), amongst which (**6**) fully eliminated the agonistic response. This was observed both for the bitter tea flavonoid ECG and for the synthetic bitter compound denatonium benzoate. The activation of hTAS2R14, another bitter receptor recognizing ECG [16], was also inhibited by the three flavanones, though to a lesser extent. A mechanistic explanation for different effects observed after simultaneous and stepwise application, remains to be established.

In view of the fact that the application of (**11**) and (**3**) did not lead to full inhibition of the ECG signal on hTAS2R39, the question arises whether these two compounds are antagonists or partial agonists. They were tested for hTAS2R39 agonism as well, but none of them activated the receptor (**File S4**). Hence, they probably act as real antagonists.

### Structural requirements for hTAS2R39 antagonists

Several flavanones similar to the antagonists identified did not show inhibitory activity towards bitter receptor hTAS2R39. It turned out that only flavanones with a methoxy group on the 6-position of the A-ring, and various B-ring configurations were able to act as antagonists of hTAS2R39, as flavanone (**5**) (substitution of flavanone crucial for inhibition), 6-hydroxyflavanone (**8**) (methoxy-substitution crucial for inhibition), 4′-methoxyflavanone (**13**) (A-ring methoxylation crucial for inhibition), and 7-methoxyflavanone (**12**) (6-position crucial for inhibition) did not show inhibitory activity. Additionally, the compound 6-methoxyflavone was unable to inhibit hTAS2R39 activation (data not shown), which indicated that absence of a double bond in the C-ring is essential for inhibition.

Amongst the antagonists identified for hTAS2R39, the difference in substitution of the B-ring determined the blocking potency. Compound (**11**), which is unsubstituted on the B-ring, showed poor blocking behavior compared to (**6**) and (**3**). It might be speculated that size, electronegativity and/or electron withdrawing effect of the B-ring substituent influences the potency of antagonists.

### Pharmacological characterization of 4′-fluoro-6-methoxyflavanone

Due to full elimination of agonistic responses (at their EC_80_ concentrations) by 4′-fluoro-6-methoxyflavanone (**6**) on hTAS2R39, this molecule was investigated further with respect to a possible antagonistic mechanism. Two effects were observed when increasing 4′-fluoro-6-methoxyflavanone concentrations: the dose-response curves shifted to the right, and the signal amplitudes decreased. These phenomena suggest that it can be classified as insurmountable antagonist [22]. This curve pattern can be an indication for three different mechanisms: (i) irreversible antagonism, (ii) reversible non-competitive orthosteric antagonism, in which an equilibrium is not reached, and (iii) reversible insurmountable allosteric antagonism [22]. Washout experiments (**Figure 8C**) clearly showed that the ECG responses after washing-out the antagonist were similar to the ECG responses prior to application of the antagonist. It can thus be concluded that the interaction was reversible. In order to distinguish between (ii) and (iii), two aspects can be studied: (a) whether the antagonist is probe dependent, meaning that its antagonistic characteristics (reflected in curve shapes) are different towards structurally different agonists, and (b) whether the effect of the antagonist is saturable, meaning that no antagonist concentration can lead to full agonist signal elimination. Probe dependence (a) and saturability (b) indicate mechanism (iii) [22]. When the inhibition behavior of 4′-fluoro-6-methoxyflavanone was studied with two structurally different agonists, it was observed that the synthetic hTAS2R39 agonist denatonium benzoate was also inhibited by 4′-fluoro-6-methoxyflavanone (**Figure 8B**), and exhibited a curve pattern almost alike that by ECG (**Figure 8A**) upon different blocker concentrations. These analogous results for the two structurally different agonists might on the one hand indicate orthosteric antagonism, but on the other hand they do not completely exclude allosteric antagonism. If the antagonist would have been unable to inhibit a structurally different agonist, it would strongly suggest allosteric antagonism. However, determination of allosterism by observing probe dependence is a one-way relationship [22], meaning that the absence of probe dependence is no definite proof for orthosterism. The same holds for saturability, which was not examined.

For hTAS2R46, hTAS2R31, hTAS2R43 [23], hTAS2R16 [24], and hTAS2R38 [25], docking simulations into homology models, validated by site-directed mutagenesis, have predicted the presence of a single binding pocket in the respective bitter receptors. Furthermore, the mechanism of antagonism of GIV3727 on hTAS2R31 was described as orthosteric, insurmountable antagonism [5], supported by docking the antagonist into the same binding pocket as the agonist. In contrast, one study suggests allosteric antagonism as mechanism for inhibition of hTAS2R16 and hTAS2R38 by probenecid [6]. No information has been reported yet on the binding pocket of hTAS2R39. As the majority of studies suggests the presence of a single binding pocket in different bitter receptors, and the relatively small extracellular domain of bitter receptors offers little space for another binding site (in contrast to sweet receptors, where a second binding site is present in the large N-terminal extracellular domain [26]), an orthosteric mechanism seems more likely for explaining our observations. However, at the antagonist concentrations tested, no full signal elimination was reached, and thus the possibility of an allosteric mechanism cannot be ruled out.

### Application of bitter receptor blockers

For application of blockers in food products, several requirements should be met. (i) The blocker should be functional at a low dose. Therefore, an antagonist that has to be applied in equimolar or higher quantity to the agonist, is not efficient. (ii) In order to block the bitter taste of dietary compounds, for practical reasons, it is necessary that a bitter receptor blocker is also functional when applied simultaneously with the bitter compound. Therefore, compounds like (**3**) and (**6**) seem to be more suitable than (**11**). (iii) In order to achieve a sensorial effect, blocking of all bitter receptors activated by one compound is desirable. A blocker, that can only inhibit hTAS2R39, but not hTAS2R14, might be too specific to effectively reduce bitterness of ECG. On the other hand, it is not known yet whether these two receptors have an equally important role in the mouth. (iv) The blocker should preferably be of natural origin, and should be available in sufficient quantity. We could not find any natural source of the three inhibitors described in this study, and assume that they are only available synthetically. (v) Only compounds that are known as safe for consumption are of interest for food applications. Due to unknown safety of 4′-fluoro-6-methoxyflavanone, we abstained from sensory tests, which might confirm the function as bitter taste blocker *in-vivo*. As not all criteria are met by the blockers discovered in the present study, they might not be applicable to food products. Nevertheless, a 6-methoxy substituent on the A-ring of a flavanone has been identified as important for inhibition of hTAS2R39, which might form the basis for other, more suitable, blockers.

## Supporting Information

File S1 **The effect of probenecid.**
(PDF)

File S2 **Test for toxic effects.**
(PDF)

File S3 **Investigation of identified inhibitors on hTAS2R16.**
(PDF)

File S4 **Investigation of agonism on hTAS2R39.**
(PDF)
