# Supplementary material for: 6-Methoxyflavanones as Bitter Taste Receptor Blockers for hTAS2R39
Source: PLoS One. 2014 Apr 10;9(4):e94451. doi: 10.1371/journal.pone.0094451 (PMC3983201; doi:10.1371/journal.pone.0094451)
Supplement: File S1 — The effect of probenecid. (PDF) [file pone.0094451.s001.pdf]

File S1 The effect of probenecid.

Probenecid is often used in bitter receptor assays (e.g. Intelmann et al., *Chemosens. Percept.* 2009, 2, 118-132; Meyerhof et al., *Chem. Senses* 2010, 35, 157-170; Slack et al., *Current Biology*, 2010, 20, 1104-1109, suppl.; Hellfritsch et al., *J. Agric. Food Chem.* 2012, 60, 6782-6793) in order to prevent efflux of calcium-sensitive fluorescent dyes from the cells (Merritt et al., *Biochem. J.* 1990, 269, 513-519). However, one article reported inhibition of activation of hTAS2R16, hTAS2R38, and hTAS2R43 by probenecid (Greene et al., *PLOS ONE*, 2011, 6, e20123). Therefore, we investigated the effect of probenecid on our receptor assay in the quantities usually used in our assay. The effect of the presence and absence of probenecid was investigated in the buffer and in the dye loading buffer on the calcium response measured.

Tyrode's buffer (containing 0.5 mM probenecid) was used for dilution of compound-DMSO stock solutions and for calcium imaging assays. Prior to measuring, the cell plates were incubated (60 min) with "dye loading buffer" (Tyrode's buffer containing 2.5 µM fluorescent calcium dye Fluo 4-AM). One hour after loading, cells were washed with Tyrode's buffer and taken up in Tyrode's buffer. Thus, according to our normal protocol, probenecid was present in the Tyrode's buffer ("buffer +") and in the dye loading buffer ("dye +"). Additionally, an adapted protocol was used without probenecid in the Tyrode's buffer ("buffer -")("dye +"), without probenecid in the dye loading buffer ("buffer +")("dye -"), and without probenecid in the both Tyrode's buffer and dye loading buffer ("buffer -")("dye -"). Calcium responses induced by the agonists equol, epicatechin gallate (ECG) and salicin were measured on the bitter taste receptors hTAS2R14, hTAS2R39, and hTAS2R16, respectively.

As can be seen in **Figures S1A-S1C**, the total absence of probenecid ("buffer -")("dye -") led to absence of calcium signals despite the presence of receptor agonists. The presence of probenecid in either the Tyrode's buffer or the dye loading buffer ("buffer +")("dye -") or ("buffer -")("dye +") led to very low calcium signals in the presence of receptor agonists. Only the presence of probenecid in both Tyrode's buffer or dye loading buffer ("buffer +")("dye +") led to acceptable calcium signals elicited by receptor agonists. Thus, no inhibitory effect of probenecid was observed, but rather it was observed that the use of probenecid was necessary to obtain acceptable signals in our assays. As the probenecid concentration used in our assays was always the same for each well of the microtiter plate, and the quantity of buffer containing probenecid was also always the same for each well, it was concluded that our observations were not influenced by possible inhibitory properties of probenecid.

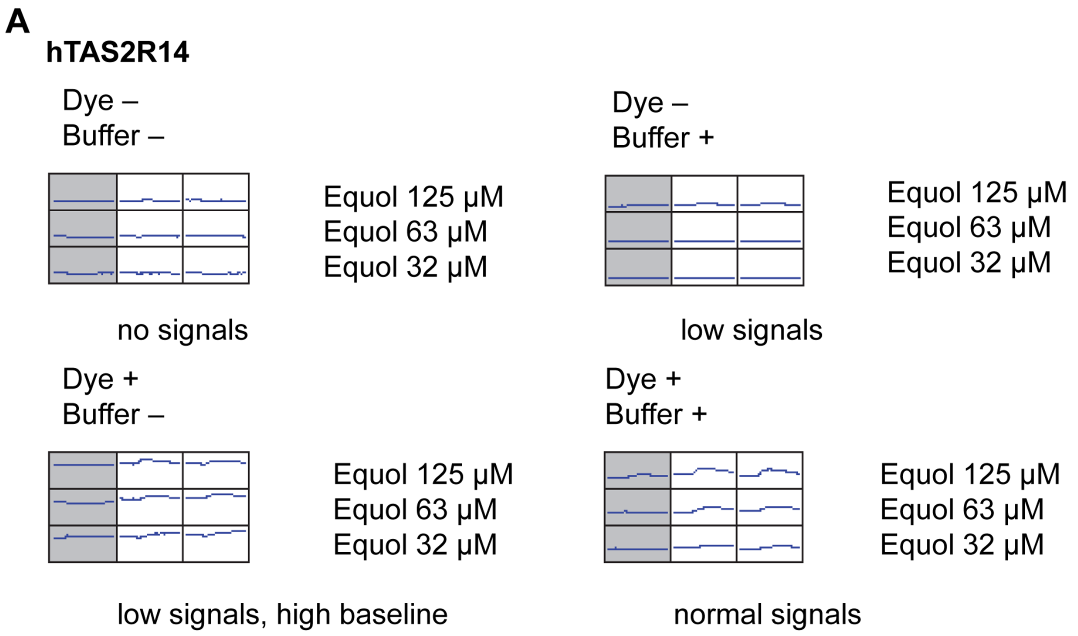

Figure S1A. Bitter receptor activation of hTAS2R14 by equol (top rows 125 μM, middle rows 63 μM, bottom rows 32 μM). The signals of induced cells (expressing bitter receptors) are depicted with white background. Non-induced cells (not expressing bitter receptors) were used as negative controls (depicted with grey background).

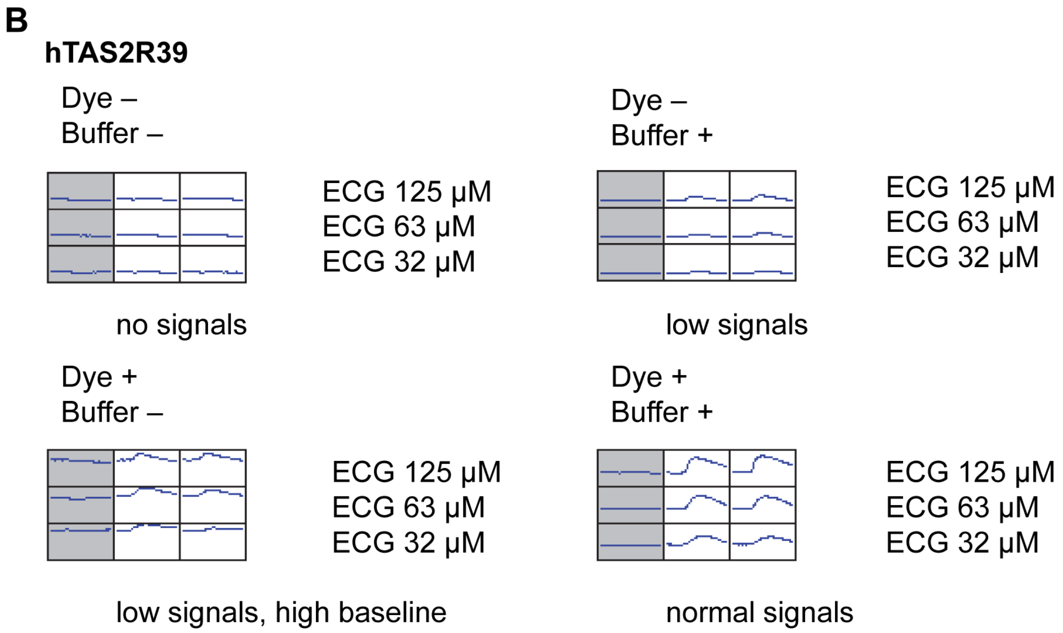

Figure S1B. Bitter receptor activation of hTAS2R39 by epicatechin gallate (ECG) (top rows 125 μM, middle rows 63 μM, bottom rows 32 μM). The signals of induced cells (expressing bitter receptors) are depicted with white background. Non-induced cells (not expressing bitter receptors) were used as negative controls (depicted with grey background).

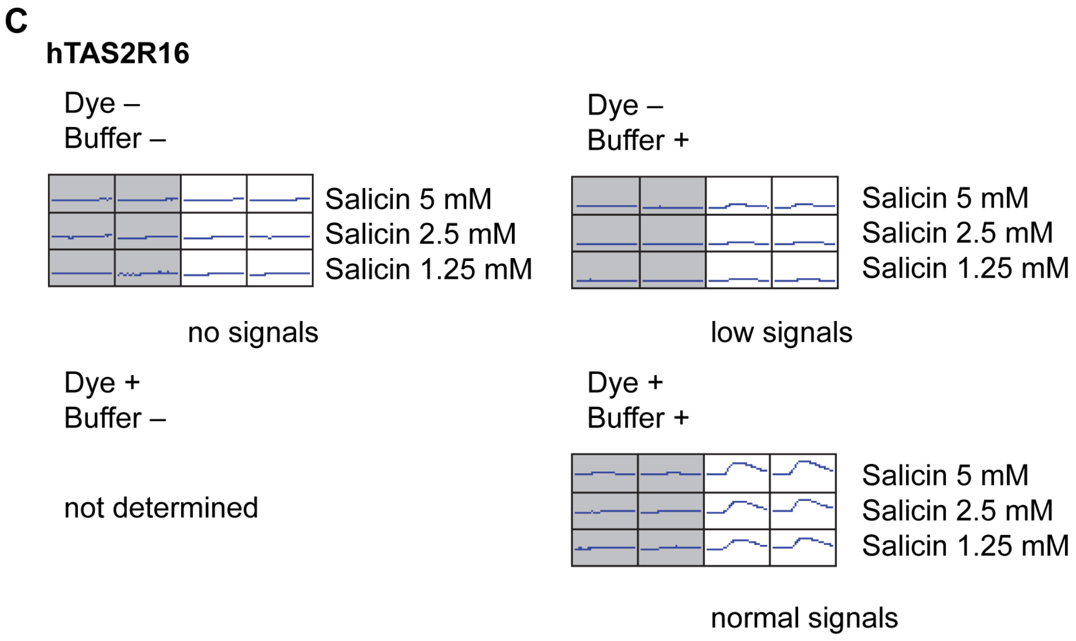

Figure S1C. Bitter receptor activation of hTAS2R16 by salicin (top rows 5 mM, middle rows 2.5 mM, bottom rows 1.25 mM). The signals of induced cells (expressing bitter receptors) are depicted with white background. Non-induced cells (not expressing bitter receptors) were used as negative controls (depicted with grey background).
