# Supplementary material for: 6-Methoxyflavanones as Bitter Taste Receptor Blockers for hTAS2R39
Source: PLoS One. 2014 Apr 10;9(4):e94451. doi: 10.1371/journal.pone.0094451 (PMC3983201; doi:10.1371/journal.pone.0094451)
Supplement: File S2 — Test for toxic effects. (PDF) [file pone.0094451.s002.pdf]

File S2 Test for toxic effects.

Compounds were tested for toxic effects on the cells by means of dye exclusion tests. The experiments were conducted as described in Roland et al., *J. Agric. Food Chem.* 2011, 59, 11764-11771. The results are shown in **Figure S2**. No toxic effects on the cells were observed.

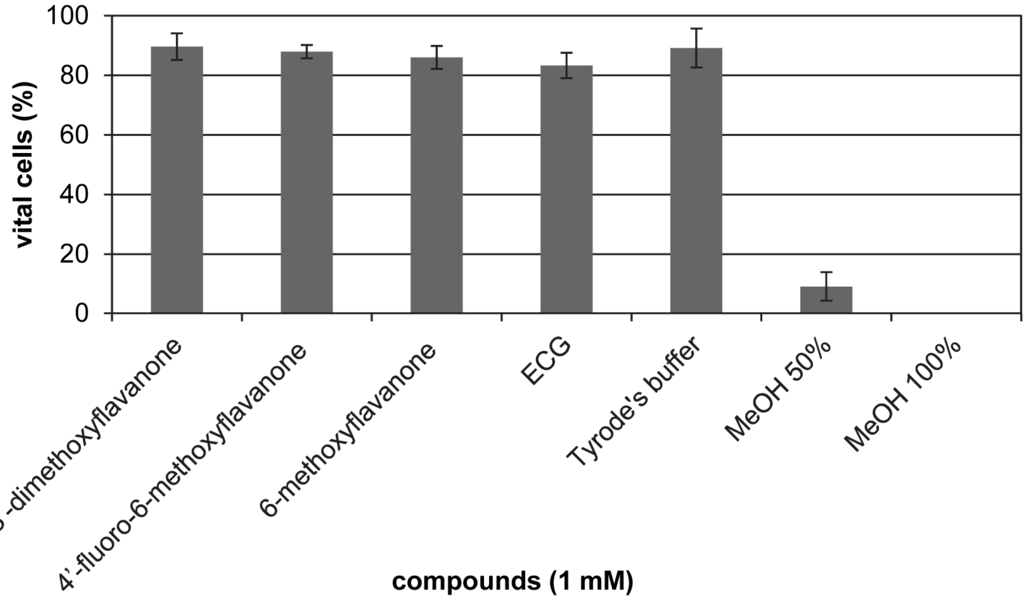

Figure S2. Cell viability after 2 min incubation with antagonists, ECG, Tyrode's buffer (negative control for toxicity), and methanol (positive control for toxicity).
