# Supplementary material for: 6-Methoxyflavanones as Bitter Taste Receptor Blockers for hTAS2R39
Source: PLoS One. 2014 Apr 10;9(4):e94451. doi: 10.1371/journal.pone.0094451 (PMC3983201; doi:10.1371/journal.pone.0094451)
Supplement: File S3 — Investigation of identified inhibitors on hTAS2R16. (PDF) [file pone.0094451.s003.pdf]

File S3 Investigation of identified inhibitors on hTAS2R16.

To investigate whether the hTAS2R39 inhibitors 6,3'-dimethoxyflavanone (**3**), 4'-fluoro-6-methoxyflavanone (**6**), and 6-methoxyflavanone (**11**) were able to inhibit a bitter receptor not known for flavonoid activation, hTAS2R16 was selected for inhibition experiments. Measuring dose-response curves of the hTAS2R16 agonist salicin under the conditions used in this study revealed an EC_{80} concentration of 10 mM salicin on hTAS2R16. The results in **Figure S3** show that no inhibition of hTAS2R16 occurred.

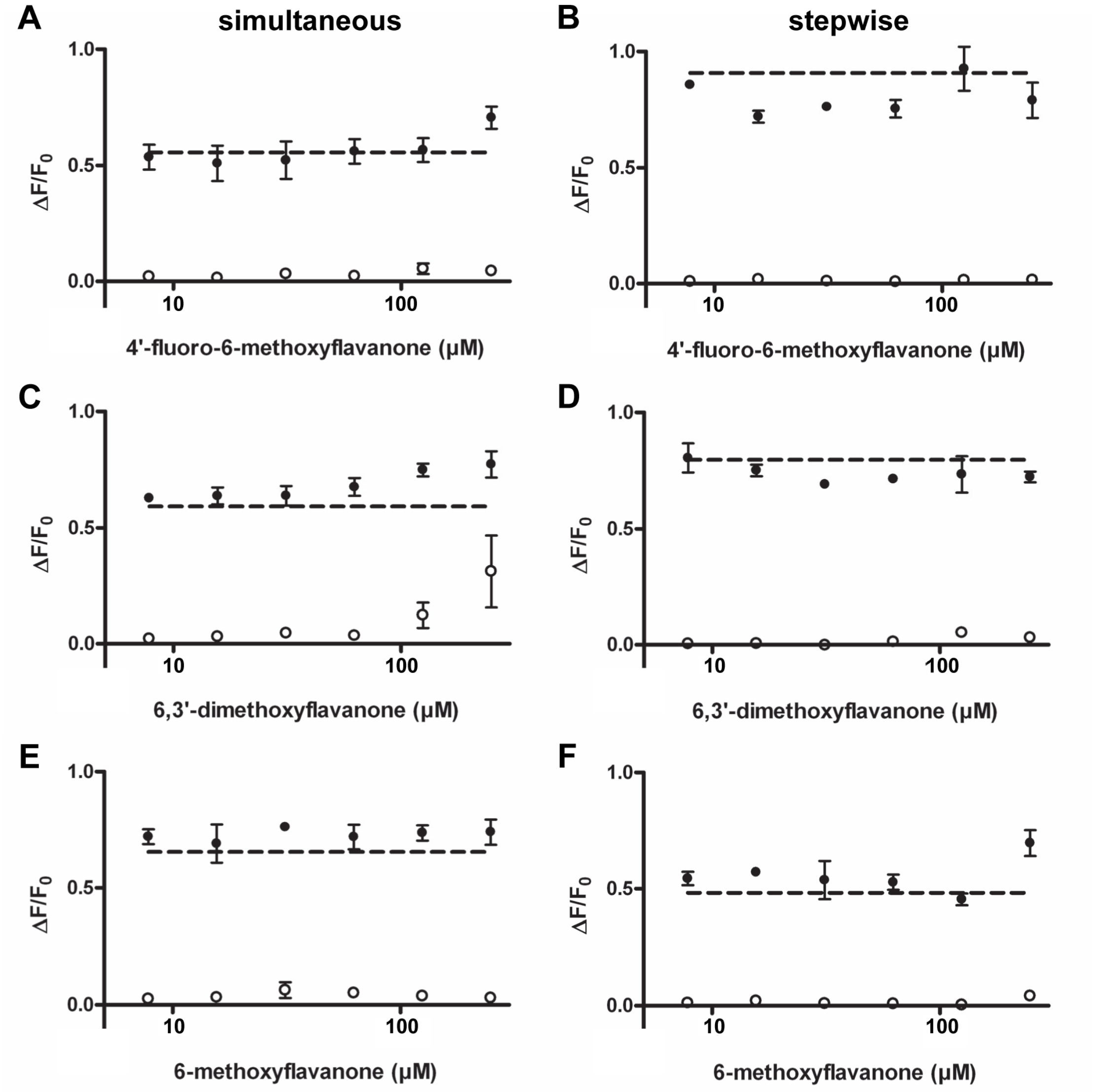

Figure S3. Inhibition of response of 10 mM salicin (---) on hTAS2R16 (induced (●), non-induced (○)) by 4'-fluoro-6-methoxyflavanone (**6**) after simultaneous addition (**A**) and stepwise addition (**B**), by 6,3'-dimethoxyflavanone (**3**) after simultaneous addition (**C**) and stepwise addition (**D**), and by 6-methoxyflavanone (**11**) after simultaneous addition (**E**) and stepwise addition (**F**).
