# Supplementary material for: 6-Methoxyflavanones as Bitter Taste Receptor Blockers for hTAS2R39
Source: PLoS One. 2014 Apr 10;9(4):e94451. doi: 10.1371/journal.pone.0094451 (PMC3983201; doi:10.1371/journal.pone.0094451)
Supplement: File S4 — Investigation of agonism on hTAS2R39. (PDF) [file pone.0094451.s004.pdf]

**File S4** Investigation of agonism on hTAS2R39.

As described in the manuscript, application of 6,3-dimethoxyflavanone (**11**) and 6-methoxyflavanone (**3**) did not lead to full inhibition of the ECG signal on hTAS2R39. In order to investigate whether these compounds might be partial agonists on hTAS2R39, they were tested for hTAS2R39 agonism. **Figures S4A and S4B** show that both compounds elicited non-specific signals at higher concentrations, but they did not activate hTAS2R39.

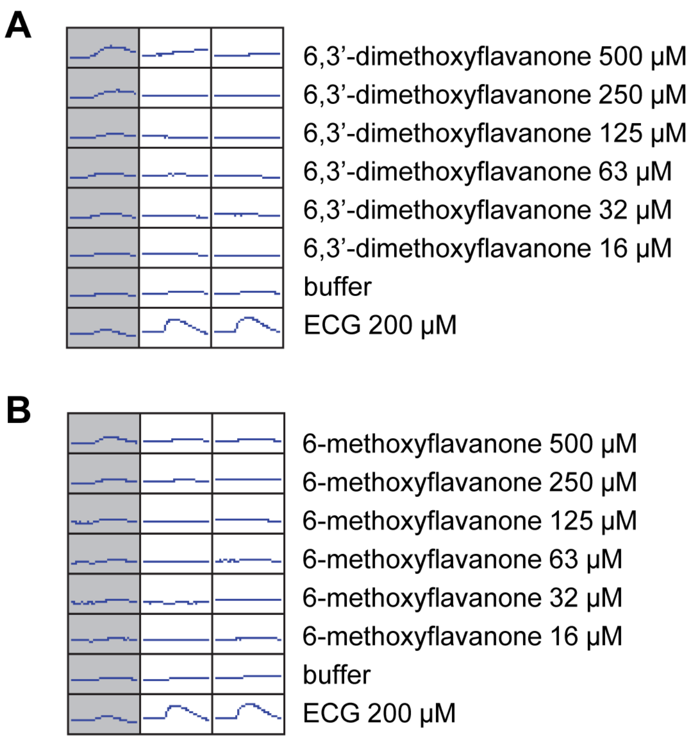


**Figure S4.** Response of hTAS2R39 towards stimulation with 6,3-dimethoxyflavanone (concentrations 500 µM – 16 µM), buffer, and ECG (**A**) and 6-methoxyflavanone (concentrations 500 µM – 16 µM), buffer, and ECG (**B**). The signals of induced cells (expressing bitter receptors) are depicted with white background. The signals of non-induced cells (not expressing bitter receptors) are depicted with grey background.
